# Prognostic Awareness and Discussions of Incurability in Patients with Pretreated Non-Small Cell Lung Cancer and Caregivers: A Prospective Cohort Study

**DOI:** 10.1093/oncolo/oyac178

**Published:** 2022-09-06

**Authors:** Takaaki Hasegawa, Toru Okuyama, Takehiro Uemura, Yoshinobu Matsuda, Hiroyuki Otani, Junichi Shimizu, Yoshitsugu Horio, Naohiro Watanabe, Teppei Yamaguchi, Satoshi Fukuda, Tetsuya Oguri, Ken Maeno, Akihiro Tamiya, Kaname Nosaki, Kensuke Fukumitsu, Tatsuo Akechi

**Affiliations:** Center for Psycho-oncology and Palliative Care, Nagoya City University Hospital, Mizuho-cho, Mizuho-ku, Nagoya, Japan; Center for Psycho-oncology and Palliative Care, Nagoya City University Hospital, Mizuho-cho, Mizuho-ku, Nagoya, Japan; Department of Psychiatry and Cognitive-Behavioral Medicine, Nagoya City University Graduate School of Medical Sciences, Mizuho-cho, Mizuho-ku, Nagoya, Japan; Department of Psychiatry, Nagoya City University West Medical Center, Kita-ku, Nagoya, Japan; Department of Respiratory Medicine, Allergy and Clinical Immunology, Nagoya City University Graduate School of Medical Sciences, Mizuho-cho, Mizuho-ku, Nagoya, Japan; Department of Thoracic Oncology, Aichi Cancer Center Hospital, Chikusa-ku, Nagoya, Aichi, Japan; Department of Psychosomatic Internal Medicine, National Hospital Organization Kinki-Chuo Chest Medical Center, Kita-ku, Sakai, Osaka, Japan; Department of Palliative Care Team, and Palliative and Supportive Care, National Hospital Organization Kyushu Cancer Center, Mitami-ku, Fukuoka, Japan; Department of Palliative Care Team, and Palliative and Supportive Care, St. Mary’s Hospital, Kurume-shi, Fukuoka, Japan; Department of Thoracic Oncology, Aichi Cancer Center Hospital, Chikusa-ku, Nagoya, Aichi, Japan; Department of Thoracic Oncology, Aichi Cancer Center Hospital, Chikusa-ku, Nagoya, Aichi, Japan; Department of Thoracic Oncology, Aichi Cancer Center Hospital, Chikusa-ku, Nagoya, Aichi, Japan; Department of Thoracic Oncology, Aichi Cancer Center Hospital, Chikusa-ku, Nagoya, Aichi, Japan; Department of Respiratory Medicine, Allergy and Clinical Immunology, Nagoya City University Graduate School of Medical Sciences, Mizuho-cho, Mizuho-ku, Nagoya, Japan; Department of Respiratory Medicine, Allergy and Clinical Immunology, Nagoya City University Graduate School of Medical Sciences, Mizuho-cho, Mizuho-ku, Nagoya, Japan; Department of Education and Research Center for Community Medicine, Nagoya City University Graduate School of Medical Sciences, Mizuho-cho, Mizuho-ku, Nagoya, Japan; Department of Respiratory Medicine, Allergy and Clinical Immunology, Nagoya City University Graduate School of Medical Sciences, Mizuho-cho, Mizuho-ku, Nagoya, Japan; Department of Internal Medicine, National Hospital Organization Kinki-Chuo Chest Medical Center, Kita-ku, Sakai, Osaka, Japan; Department of Thoracic Oncology, National Hospital Organization Kyushu Cancer Center, Minami-ku, Fukuoka-shi, Fukuoka, Japan; Department of Thoracic Oncology, National Cancer Center Hospital East, Kashiwa-shi, Chiba, Japan; Department of Respiratory Medicine, Allergy and Clinical Immunology, Nagoya City University Graduate School of Medical Sciences, Mizuho-cho, Mizuho-ku, Nagoya, Japan; Center for Psycho-oncology and Palliative Care, Nagoya City University Hospital, Mizuho-cho, Mizuho-ku, Nagoya, Japan; Department of Psychiatry and Cognitive-Behavioral Medicine, Nagoya City University Graduate School of Medical Sciences, Mizuho-cho, Mizuho-ku, Nagoya, Japan

**Keywords:** neoplasms, lung cancer, awareness, end-of-life discussion, communication, advance care planning, palliative care

## Abstract

**Background:**

Although patients with advanced cancer often have poor prognostic awareness, the most effective communication approach for improving prognostic awareness is unclear. In addition, the association between prognostic awareness and preferences for future medical treatment remains unexplored.

**Materials and Methods:**

We performed a prospective observational study of consecutive patients with advanced or post-operative recurrent non-small cell lung cancer whose disease had progressed after first–line chemotherapy, and their caregivers. We evaluated patterns of clinical discussions about incurability, prognostic awareness, and preference for future medical treatment at baseline and 3 months later.

**Results:**

We obtained 200 valid responses to the questionnaires at baseline and 147 valid responses 3 months later. In addition, 180 caregivers returned valid responses. A total of 54% of patients and 51% of caregivers had accurate awareness at baseline, and 52% of patients had accurate awareness 3 months later. Multiple logistic regression analysis revealed that patients who were informed about incurability in recent and past discussions were significantly more likely to have accurate awareness 3 months later, compared with those who were only informed recently (adjusted odds ratio 5.08; 95% CI, 1.31-19.78; *P* = .019). Accurate awareness at 3 months was significantly negatively associated with preference for life-prolonging treatment at 3 months after adjusting for covariates (adjusted odds ratio 0.39; 95% CI, 0.17-0.90; *P* = .028).

**Conclusion:**

Patients with advanced cancer who had both recent and past discussions about incurability with their oncologists have more accurate prognostic awareness. Improving prognostic awareness could reduce the preference for life-prolonging treatment.

## Teaser

Developing accurate prognostic awareness promotes optimal quality of end-of-life care; however, patients with advanced cancer often have poor prognostic awareness. This article evaluates the effect of frequency of clinical discussions about incurability on the prognostic awareness of patients with advanced non-small cell lung cancer and of their caregivers.

**Implications for Practice**

In this longitudinal observational multicenter study in patients with non-small cell lung cancer and their caregivers, we found that patients with repeated clinical discussion of incurability were more likely to have accurate prognostic awareness and that patients with accurate awareness were significantly less likely to have a preference for life-prolonging treatment. Our results indicated that oncologists should evaluate patients’ and caregivers’ prognostic awareness repeatedly and continue to encourage discussion on this issue as necessary. Such efforts may help to reduce the number of patients with a preference for life-prolonging treatment.

## Introduction

Accurate prognostic awareness in patients with advanced cancer, comprising knowledge about their prognosis that includes the terminal nature of illness, life-expectancy, and incurability,^1^ is fundamental for engaging patients in end-of-life discussion.^2^ Developing accurate prognostic awareness promotes optimal quality of end-of-life care.^3,4^ Patients’ awareness about their terminal illness can have a beneficial effect on goal concordance care.^5^

However, patients with advanced cancer often have poor prognostic awareness. A meta-analysis reported that less than half of patients with advanced cancer accurately understood their prognosis.^6^ A recent systematic review reported that prognostic awareness is a complex phenomenon associated with various positive and negative factors.^7^ There is currently a lack of effective interventions to improve prognostic awareness,^8^ and interventions to promote prognostic discussion have failed to improve prognostic awareness. Although patients often have a desire to understand their prognosis,^9^ many oncologists are hesitant to discuss end-of-life care because they are worried about the potential impact on patients’ psychological status.^10^ Further research is needed to determine how to more effectively communicate with patients to promote understanding of illness.

Recent studies reported that the provision of prognostic information is a key component for developing accurate prognostic awareness, and that ongoing end-of-life discussions with oncologists can result in better prognostic awareness in patients with advanced cancer.^1,7,11^ However, because this study involved methodological limitations such as a cross-sectional design, as well as mixed cancer types and stages, the evidence supporting this conclusion is insufficient. The current study sought to evaluate the effects of the frequency of occurrence of clinical discussions about incurability on prognostic awareness in patients with advanced non-small cell lung cancer (NSCLC) who had recently experienced a failure of first-line chemotherapy. We selected patients with pretreated NSCLC because this condition is considered to be incurable^12^ and lung cancer is a leading cause of mortality from cancer,^13^ with NSCLC being responsible for the largest number of deaths among patients with lung cancer.

In addition, we aimed to investigate prognostic awareness in caregivers of patients. Few studies have examined prognostic understanding among caregivers, or the consistency between patients’ and caregivers’ awareness in east Asian countries in which family-centered decision making is conducted.^14^

In addition, many terminally ill patients with cancer receive invasive treatment until just before death.^15^ Even if invasive treatment immediately before death is in accord with the patient’s preference for future treatment, it is uncertain whether patients’ preferences are based on accurate prognostic awareness. Few previous studies have focused on the association between prognostic awareness and preferences for future medical treatment. Therefore, in the current study, we also explored the association between accurate prognostic awareness and the preference for life-prolonging medical treatment.

## Materials and Methods

### Study Design

This multicenter prospective cohort study was conducted at 4 Japanese study sites (Nagoya City University Hospital, Aichi Cancer Center Hospital, National Hospital Organization Kinki-Chuo Chest Medical Center, and the Kyushu Cancer Center). Consecutive patients attending medical follow-up appointments with participating oncologists and their caregivers were screened between 8 May 2017 and 15 March 2021. Both outpatients and inpatients were included. In addition, we recruited the caregivers identified by the patients as their primary caregiver. The study was approved by the institutional review board at each site, and was conducted in accordance with the principles of the Declaration of Helsinki. All participants were provided with a detailed description of the purpose and methods of this study before being asked to complete the questionnaires. Written informed consent was obtained from all participants. This study is registered with the University Hospital Medical Information Network (UMIN000026436).

### Patients and Their Caregivers

The present study included patients with advanced NSCLC whose disease had progressed after first-line chemotherapy (cytotoxic agents with or without immune checkpoint inhibitor). The participants included NSCLC patients with targetable genetic aberrations (such as EGFR mutations, ALK rearrangements, ROS-1, and BRAF), whose disease progressed after chemotherapy (cytotoxic agents with or without immune checkpoint inhibitors) and whose prior therapy included molecular targeted therapy.

Inclusion criteria for patients were as follows: diagnosis of stage IIIB not amenable to curative treatment, stage IV, or post-operative recurrent NSCLC, age ≥20 years, within 2 months after failure of first-line chemotherapy, and ability to understand written or spoken Japanese. Exclusion criteria were as follows: too ill to complete a questionnaire, severe mental disorders, severe cognitive disorders, or judged by the treating physician to be unsuitable for participation. Inclusion criteria for the caregivers were as follows: patients participated in this study, age ≥20 years, and ability to understand written or spoken Japanese. The exclusion criteria were the same as those for patients.

## Measurement

### Perceptions of Illness and Goals of Current Therapy

The instructions at the beginning of the questionnaire began with the following sentence: “Hereafter, we would like to ask your thoughts regarding your current illness.” Patients were then asked “How would you describe your current health condition?” with the following response options: “Good. There is hope that I will recover,” “Serious. But there is hope that I will recover,” “Good. But there is no hope that I will recover,” “Serious. There is no hope that I will recover,” “I don’t know,” or “I don’t wish to answer,” with reference to previous studies.^3,16^ Patients who responded “Good. But there is no hope that I will recover,” and “Serious. There is no hope that I will recover” were coded as having “accurate awareness.” Patients who responded “Good. There is hope that I will recover,” “Serious. But there is hope that I will recover,” “I don’t know,” and “I don’t wish to answer” were coded as “inaccurate awareness.”^12^ Patients were classified on the basis of longitudinal changes in illness perception from baseline (within 2 months after failure of first-line chemotherapy) to 3 months later. Patients were classified as follows: “remained accurate,” “became accurate,” “became inaccurate,” and “remained inaccurate.”

To assess patients’ perceptions of their current therapy goals, we asked patients how likely they thought the chemotherapy they were currently receiving was to “extend your life,” “completely cure your cancer,” or “reduce your cancer-related symptoms.”^4,16^ The response options were “absolutely agree,” “agree,” “unsure,” “disagree,” and “absolutely disagree.” Patients were asked to complete these questions at baseline and 3 months later.

The question regarding patients’ current health condition was also asked of caregivers at baseline.

### Preference for Future Medical Treatment

To assess preferences for future medical treatment, patients were asked “If you could choose, would you prefer, when there is no indication for chemotherapy” with the response options “a course of treatment that focused on extending life as much as possible, even if that meant more pain and discomfort?” or “a plan of care that focused on relieving pain and discomfort, even if that meant not living as long?”^3^ Patients who chose the former were designated as preferring life-prolonging treatment, and those who chose the latter were designated as preferring symptom-directed treatment.

### Patient Perceptions of Oncologist Disclosure of Incurability

At baseline, patients were asked “Have you ever discussed the incurability of your cancer with a physician?” At 3 months later, patients were asked “Have you discussed the incurability of your cancer with a physician in the past 2 months?” Response options for each of these questions were “yes” or “no.” Patterns of discussion were classified on the basis of reported clinical discussion from baseline to 3 months later. Patterns of discussion were categorized as follows: only recent (in the last 2 months), only in the past (prior to baseline), both recent and in the past, or incurability has never been discussed with the oncologist.^11^ We use the word “pattern” to refer to the frequency and time of occurrence of the discussions.

## Covariates

### Depressive Symptoms

The Patient Health Questionnaire-9 (PHQ-9) was administered to assess patients’ depressive symptoms.^17^ Total scores on the PHQ-9 range from 0 to 27, with higher scores indicating more severe depressive symptoms. The PHQ-9 has been validated in the Japanese population.^18^ The optimal cutoff point for screening for major depressive disorders in Japanese patients is reported to be 9/10.

### QOL

The Comprehensive Quality of Life Outcome (CoQoLo) inventory was administered to assess patients’ comprehensive quality of life (QOL).^19^ The CoQoLo is based on the concept of a good death for patients with advanced cancer. In this study, we used the short version of the CoQoLo, which consists of 18 items and has sufficient reliability and validity. Higher scores indicate better QOL.

### Physician Compassion

The Physician Compassion Questionnaire (PCQ) consists of 5 dimensions: warm/cold, pleasant/unpleasant, compassionate/distant, sensitive/insensitive, and caring/uncaring. Each dimension is scored on a scale of 0-10. The sum of the 5 scales gives a final score of 0-50. Lower scores indicate greater physician compassion (0 = best, 50 = worst).^20,21^

## Data Collection

Patients and their caregivers completed a baseline questionnaire including demographic characteristics, within 2 months after the progressive disease of first-line chemotherapy (Supplementary Table S1). We administered the follow-up assessments for patients 3 months later (or at a clinic visit within 4 weeks of that time). Follow-up assessments included illness perception, goals of therapy, CoQoLo, PHQ-9, and preference for future medical treatment. We also asked patients whether they discussed the incurability of their cancer with their physician during the last 2 months at follow-up assessment. The researchers contacted participants in cases of unanswered data. We obtained patient characteristics, including age and gender, from medical records. Performance status and histology of lung cancer were obtained from oncologists at baseline.

## Statistics

Analyses began with descriptive summaries of demographic and clinical variables. In addition, we summarized the reported illness perception and perceptions of the goals of therapy. We explored longitudinal perceptions of accurate awareness. Inter-rater agreement for patient and caregiver prognostic awareness at baseline, and inter-rater agreement for patients’ prognostic awareness at baseline and at 3 months later were evaluated using the kappa statistic. To investigate the association between accurate awareness at 3 months later and oncologist disclosure of incurability, logistic regression models were used with the following covariates: age (years), gender (female), education level (≥high school), performance status, depressive symptoms (PHQ-9 ≥ 10), QOL (CoQoLo score), and physician compassion (PCQ score). These covariates were obtained from baseline data. In addition, to investigate the association between preference for future medical treatment at 3 months later and accurate awareness, logistic regression models were used with the following covariates: age (years), gender (female), education level (≥high school), caregiver’s accurate awareness, depressive symptoms (PHQ-9 ≥10), and QOL (CoQoLo score). Patients’ illness perception, depressive symptoms and QOL were obtained 3 months later. Covariates in these logistic regression models were selected on the basis of previous studies.^3,7,8,22,23^

*P* values of <.05 were regarded as being statistically significant. Statistical analyses were performed using SPSS v.28.0 (IBM Corp., Armonk, NY).

## Results

### Characteristics

**Figure 1.**
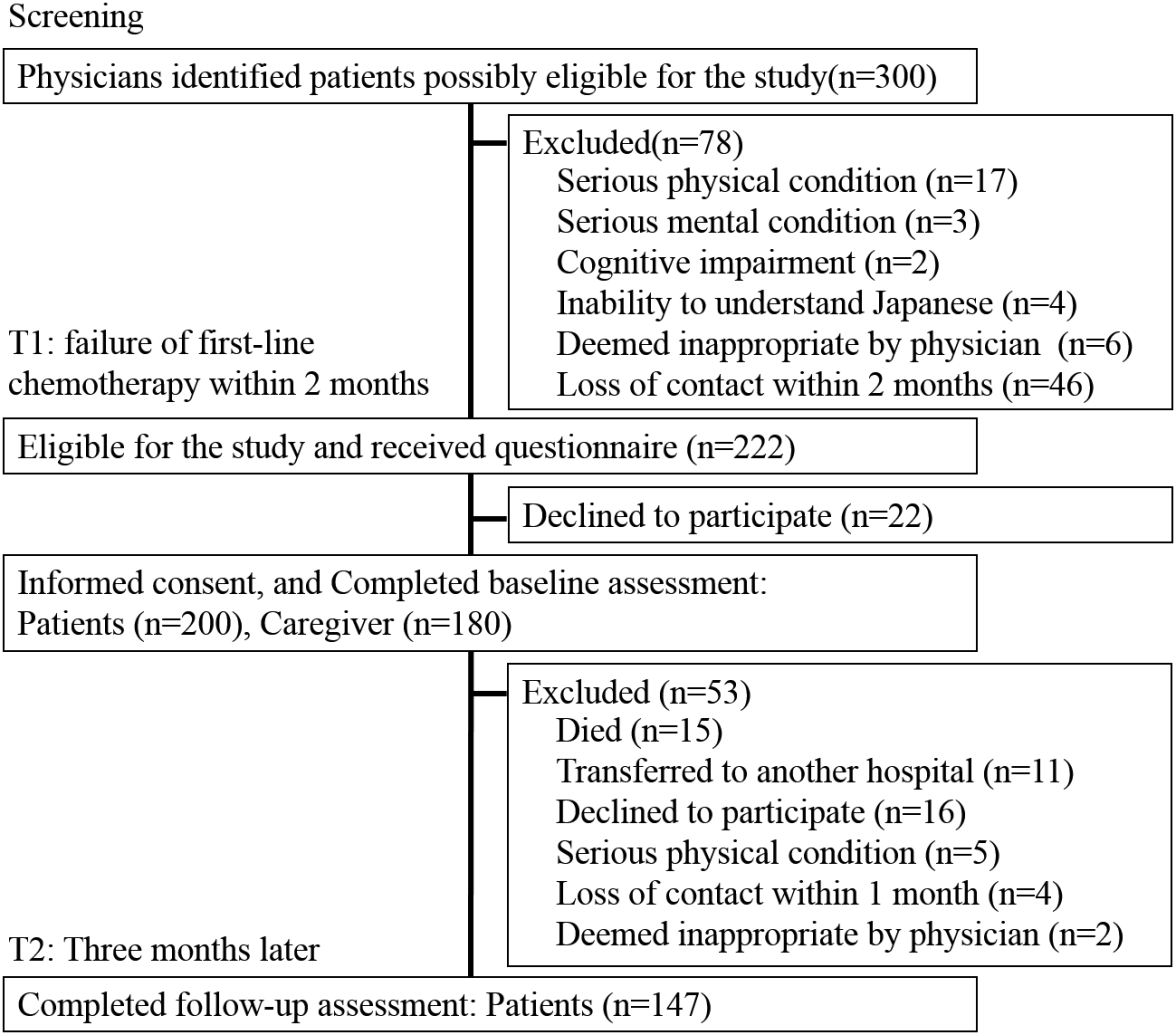
Flow chart showing recruitment of participants and follow-up.

During the study period, 300 potential participants were identified, 222 of whom were eligible, and 200 of whom returned valid questionnaire responses (Fig. 1). The number of patients that were approached and the number of refusals at each site were as follows: 47 and 3, respectively, at Nagoya City University Hospital, 144 and 13, respectively, at Aichi Cancer Center Hospital, 24 and 5, respectively, at National Hospital Organization Kinki-Chuo Chest Medical Center, and 7 and 1, respectively, at the Kyushu Cancer Center. We received valid responses from 180 caregivers. Valid responses 3 months later were obtained from 147 patients. Tables 1 and 2 summarize participants’ and caregivers’ characteristics, respectively.

**Table 1.** Patients’ characteristics

Characteristics	Baseline (within 2 months after first-line failure)				3 months later			
	Total, *n* (%)	Accurate awareness, *n* = 108 *n* (%)	Inaccurate awareness, *n* = 92 *n* (%)	*P*-value	Total, *n* (%)	Accurate awareness, *n* = 76 *n* (%)	Inaccurate awareness, *n* = 71 *n* (%)	*P*-value
Age (years)								
Mean (SD)	65.1 (10.0)	64.2 (9.9)	66.1 (10.2)	.17	65.4 (9.9)	64.9 (10.5)	66.1 (9.2)	.48
Gender								
Male	137 (68.5)	74 (68.5)	63 (68.5)	1.00	102 (69.4)	53 (69.7)	49 (69.0)	.92
Female	63 (31.5)	34 (31.5)	29 (31.5)		45 (30.6)	23 (30.3)	22 (31.0)	
Marital status								
Married	158 (79.0)	84 (77.8)	74 (80.4)	.65	121 (82.3)	60 (78.9)	61 (85.9)	.26
Other	42 (21.0)	24 (22.2)	18 (19.6)		26 (17.7)	16 (21.1)	10 (14.1)	
Education								
≥High school	161 (80.5)	91 (84.3)	70 (76.1)	.19	124 (84.4)	69 (90.8)	55 (77.5)	.026
<High school	38 (19.0)	17 (15.7)	21 (22.8)		23 (15.6)	7 0 (9.2)	16 (22.5)	
Occupation								
Paid or self-employed (full-time)	31 (15.5)	19 (17.6)	12 (13.0)	.46	25 (17.0)	15 (19.7)	10 (14.1)	.27
Paid employee (part-time)	15 (7.5)	6 (5.6)	9 (9.8)		12 (8.2)	4 (5.3)	8 (11.3)	
Homemaker	29 (14.5)	15 (13.9)	14 (15.2)		21 (14.3)	10 (13.2)	11 (15.5)	
Retirement	100 (50.0)	52 (48.1)	48 (52.2)		77 (52.4)	38 (50.0)	39 (54.9)	
Other	24 (12.0)	16 (14.8)	8 (8.7)		12 (8.2)	9 (11.8)	3 (4.2)	
Living condition								
Living with someone	175 (87.5)	94 (87.0)	81 (88.0)	.83	131 (89.1)	68 (89.5)	63 (88.7)	.89
Living alone	25 (12.5)	14 (13.0)	11 (12.0)		16 (10.9)	8 (10.5)	8 (11.3)	
PS								
0	72 (36.0)	35 (32.4)	37 (40.2)	.61	62 (42.2)	29 (38.2)	33 (46.5)	.44
1	117 (58.5)	70 (64.8)	47 (51.1)		81 (55.1)	46 (60.5)	35 (49.3)	
2	11 (5.5)	3 (2.8)	8 (8.7)		4 (2.7)	1 (1.3)	3 (4.2)	
Histology								
Non-Sq	150 (75.0)	83 (76.9)	67 (72.8)	.51	112 (76.2)	59 (77.6)	53 (74.6)	.67
Sq	50 (25.0)	25 (23.1)	25 (27.2)		35 (23.8)	17 (22.4)	18 (25.4)	

Some items did not reach the total number because of missing values.

Abbreviations: PS, Eastern Cooperative Oncology Group performance status; Sq, squamous cell carcinoma.

**Table 2.** Caregivers’ characteristics

	Baseline (within 2 months after first-line failure)			*P*-value
	Total, *n* (%)	Accurate awareness, (*n* = 92) *n* (%)	Inaccurate awareness, (*n* = 88) *n* (%)	
Age (years)				
Mean (SD)	58.8 (13.2)	56.8 (13.4)	61 (12.7)	.031
Gender				
Male	63 (35.0)	31 (33.7)	31 (35.2)	.91
Female	116 (64.4)	59 (64.1)	57 (64.8)	
Relationship with patient				
Spouse	125 (69.4)	65 (70.7)	60 (68.2)	.003
Child	34 (18.9)	23 (25.0)	11 (12.5)	
Parent	8 (4.4)	0 (0.0)	8 (9.1)	
Sibling	8 (4.4)	3 (3.3)	5 (5.7)	
Other	5 (2.8)	1 (1.1)	4 (4.5)	
Education				
≥High school	160 (88.9)	86 (93.5)	74 (84.1)	.068
<High school	19 (10.6)	6 (6.5)	13 (14.8)	
Occupation				
Paid or self-employed (full-time)	46 (25.6)	28 (30.4)	18 (20.5)	.12
Paid employee (part-time)	33 (18.3)	18 (19.6)	15 (17.0)	
Homemaker	59 (32.8)	31 (33.7)	28 (31.8)	
Retirement	30 (16.7)	9 (9.8)	21 (23.9)	
Other	12 (6.7)	6 (6.5)	6 (6.8)	

Some items did not reach the total number because of missing values.

### Illness Perception and Goals of Current Therapy

**Figure 2.**
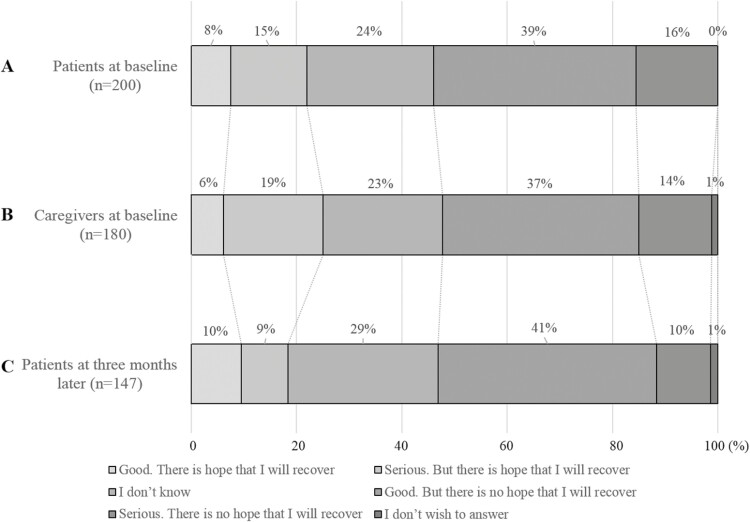
Illness perception in patients with non-small cell lung cancer and their caregivers. Patients and their caregivers were asked “How would you describe your (patient’s) current health condition” with the following response options: (**A**) Patients’ illness perception at baseline (within 2 months after first-line chemotherapy failure). (**B**) Caregivers’ illness perception at baseline. (**C**) Patients’ illness perception 3 months later.

As shown in Fig. 2, 108 (54.0%) patients had accurate awareness at baseline (95% CI, 47.1-60.8), 92 (51.1%) caregivers had accurate awareness at baseline (95% CI, 43.8-58.3), and 76 (51.7%) patients had accurate awareness 3 months later (95% CI, 43.7-59.7). The level of prognostic awareness remained stable during the 3 months over the repeated measurements (Supplementary Fig. S1). Patient/caregiver prognostic awareness agreement at baseline was fair (*κ* = 0.34, *P* < .001). Agreement between patients’ prognostic awareness at baseline and 3 months later was moderate (*κ* = 0.59, *P* < .001).

Patient-reported goals of current chemotherapy for the outcomes of cure, life extension, and symptom relief are shown in Supplementary Fig. S2. The numbers and proportions of patients with accurate perception (ie, disagree or absolutely disagree that the chemotherapy they were currently receiving would completely cure their cancer) were 84 (42.0%, 95% CI, 35.3-48.9) at baseline and 70 (47.6%, 95% CI, 39.5-55.7) at 3 months later.

### Association Between Illness Perception and Oncologist Disclosure of Incurability

Multiple logistic regression analysis revealed that patients who were informed about incurability both recently and in the past were more likely to have accurate awareness 3 months later compared with those who were informed only recently, after adjusting for gender, age, education level, performance status, depressive symptoms, QOL, and physician compassion (Table 3).

**Table 3.** Accurate prognostic awareness 3 months later by patients’ perception of disclosure of incurability

Disclosure of incurability	*N*	Unadjusted odds	95% CI	Adjusted^a^ Odds	95% CI	Adjusted^a^*P*-value
Only recent	15	Reference		Reference		-
Only past	45	2.50	0.74-8.50	2.75	0.68-11.07	.15
Both recent and past	54	4.35	1.29-14.71	5.08	1.31-19.78	.019
Never	32	0.78	0.21-2.93	0.80	0.19-3.43	.76

Logistic regression models were used to assess the association between prognostic awareness (accurate/inaccurate) 3 months later and oncologist disclosure of incurability.

^a^ Adjusted for age (years), gender, education level (≥high school), performance status, patient health questionnaire 9 (≥10), comprehensive quality of life outcome and physician compassion questionnaire

### Preference for Future Medical Treatment

**Figure 3.**
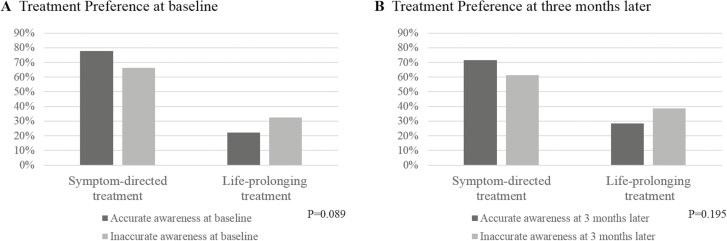
Preference for future medical treatment according to prognostic awareness. Patients were asked “If you could choose, would you prefer, when there is no indication for chemotherapy” with the response options “a course of treatment that focused on extending life as much as possible, even if that meant more pain and discomfort?” or “a plan of care that focused on relieving pain and discomfort, even if that meant not living as long?” Patients who chose the former were designated as preferring life-prolonging treatment, and those who chose the latter were designated as preferring symptom-directed treatment. Regarding illness perception, patients were asked “How would you describe your current health condition” with response options. Patients responding “Good. But there is no hope that I will recover,” and “Serious. There is no hope that I will recover” were coded as having “accurate awareness.” (**A**) Patients’ preference for future medical treatment at baseline (within 2 months after first-line chemotherapy failure). (**B**) Patients’ preference for future medical treatment 3 months later.

The number of patients with a preference for life-prolonging treatment was 54 (27%, 95% CI, 21-34) at baseline, and 48 (33%, 95% CI, 25-41) 3 months later (Fig. 3). Multiple logistic regression analysis revealed that patients with accurate awareness 3 months later were significantly less likely to have a preference for life-prolonging treatment at 3 months later than those with inaccurate awareness (Table 4).

**Table 4.** Preference for life-prolonging treatment 3 months later by patients’ prognostic awareness

Prognostic awareness	n	Unadjusted odds	95% CI	Adjusted^a^ odds	95% CI	Adjusted^a^*P*-value
Inaccurate	70	Reference		Reference		-
Accurate	74	0.631	0.314-1.268	0.39	0.17-0.90	.028

Logistic regression models were used to assess the association between preference for future treatment (life-prolonging treatment/symptom-directed treatment) 3 months later and accurate awareness 3 months later.

Adjusted for age (years), gender, education level (≥ high school), caregiver’s accurate awareness, patient health questionnaire 9 (≥10) and comprehensive quality of life outcome.

## Discussion

To the best of our knowledge, this study is one of the few novel prospective cohort surveys to explore the association between ongoing end-of-life discussions and prognostic awareness, as well as the association between prognostic awareness and preferences for future medical treatment in patients with advanced or postoperative recurrent NSCLC. The current study has several strengths. First, the study cohort comprised consecutive eligible patients at multiple study sites, and the rate of refusal was within acceptable limits. In addition, because we recruited patients with NSCLC within 2 months after failure of first-line chemotherapy, the sample was relatively homogenous. All participants were treated with second-line therapy at baseline.

The first major finding was that patients with pretreated advanced NSCLC who had continuous discussions about incurability with their oncologist tended to have accurate awareness. A previous cross-sectional study of patients with advanced cancer including mixed cancer types, reported an association between prognostic awareness and prognosis discussion patterns.^11^ We replicated the findings of previous studies with more robust study designs, including those with more homogeneous samples and longitudinal study designs. These results suggest that oncologists should communicate with patients as frequently as appropriate. Communication is valuable for filling the gap between patients’ prognostic awareness and life expectancy, while confirming patients’ desire to be informed about their prognosis. This result is consistent with previous literature on advance care planning.^24^ In some situations, an oncologist might incorrectly assume that their patient has understood the information given to them, including disclosure of incurability. In addition, oncologists tend to avoid frequent communication, because of concerns that continuously discussing the prognosis may repeatedly induce a psychological burden for patients. However, the current results indicated that oncologists should repeatedly evaluate patients’ and caregivers’ prognostic awareness, and continue to encourage discussion of this issue as necessary. These findings partially support previous reports, suggesting that early palliative care with continuous involvement of a palliative care team and repeated end-of-life discussion can improve patients’ understanding of their prognosis.^25-27^ This evidence suggests that continuous end-of-life discussion may promote accurate prognostic awareness.

The second major finding of the present study was that the preference for life-prolonging treatment was associated with inaccurate prognostic awareness. Cardiopulmonary resuscitation has a low success rate in patients with advanced cancer.^28^ The outcomes of patients with advanced cancer after intensive care unit admission are poor, and few patients regain their previous functional status.^29^ Life-prolonging treatment in end-of-life care is often aggressive, and avoidance of life-prolonging treatment is generally considered to be appropriate for patients with advanced cancer and no indication for chemotherapy. Preference for life-prolonging treatment is reported to be negatively associated with end-of-life care.^4^ However, few studies investigated the association between treatment preference in end-of-life care and illness perception.^3,22,23^ There are conflicting views regarding whether correct information by itself is sufficient to enable patients to make appropriate medical decisions.^7,8^ Importantly, the current study revealed that accurate prognostic awareness led patients to undertake appropriate decision making. Efforts to improve patients’ prognostic awareness may have served to reduce the proportion of patients who expressed a preference for life-prolonging treatment. However, we did not examine how physicians conveyed prognostic information to patients in this study. Communication about prognosis between oncologists and patients with cancer is a complex phenomenon. Integrating existing clinical research with communication research requires an interdisciplinary approach that combines medical oncology with cognitive and linguistic research.^30-32^ Future research is needed to determine how to more effectively communicate with patients to promote understanding of illness. We propose that an interdisciplinary approach that combines medical oncology with cognitive and linguistic research is a promising avenue for future research.

It should be noted that agreement between the patient and the caregiver at baseline was relatively low in the current study. The findings of our evaluation within 2 months after first-line failure are consistent with previously reported results in patients with newly diagnosed lung cancer and their caregivers.^33^ Patients and caregivers might interpret physicians’ explanations differently from diagnosis to clinical course. Patients’ and caregivers’ prognostic awareness should be evaluated individually and continuously.

It is currently unclear whether accurate prognostic awareness or advanced care planning cause psychological burden in patients.^7,34,35^ In the current study, we did not evaluate the association between prognostic awareness and QOL or mood, because these were used as covariates in statistical analysis. We plan to report on this topic in a further study in the near future.

The present study involved several limitations that should be considered. First, the questionnaire used to assess the illness perception, goals of therapy, perceptions of oncologist disclosure of incurability, and preference for future medical treatment was not fully validated. However, these questions were developed on the basis of previous studies,^3,11,16^ and their face validity was confirmed in a pilot test with 5 patients with cancer. Second, the present study was a prospective cohort study, not an intervention trial. As such, the association between patterns of discussions of prognosis and prognostic awareness revealed by this study should be confirmed in randomized controlled trials. Third, recall bias may have affected responses to the question regarding the perception of oncologist disclosure of incurability. Fourth, we investigated preferences for future treatment but did not examine the actual treatments received. We plan to examine this issue in a future study. Fifth, no formal sample calculation was performed in this study. We assumed that the sample size of 200 patients with NSCLC was adequate to investigate the association between prognostic awareness and oncologist disclosure of incurability using multiple logistic regression analysis. Finally, because the study settings were restricted, the findings cannot necessarily be generalized to other situations, such as other cancer types or clinical stages.

## Conclusion

Approximately half of the patients with pretreated advanced NSCLC recognized the incurability of their condition, and ongoing discussions about incurability were associated with accurate prognostic awareness. In addition, a preference for life-prolonging treatment was associated with inaccurate prognostic awareness. Our findings suggest that additional research is needed to identify whether ongoing prognostic disclosure is effective for improving prognostic awareness and reducing the aggressiveness of end-of-life care.

## Supplementary Material

oyac178_suppl_Supplementary_Table_S1

oyac178_suppl_Supplementary_Figure_S1

oyac178_suppl_Supplementary_Figure_S2

## Data Availability

The data underlying this article will be shared on reasonable request to the corresponding author.
