# Supplementary material for: Prognostic Awareness and Discussions of Incurability in Patients with Pretreated Non-Small Cell Lung Cancer and Caregivers: A Prospective Cohort Study
Source: Oncologist. 2022 Sep 6;27(11):982–90. doi: 10.1093/oncolo/oyac178 (PMC9632306; doi:10.1093/oncolo/oyac178)
Supplement: oyac178_suppl_Supplementary_Table_S1 [file oyac178_suppl_supplementary_table_s1.docx]

Table S1. List of questionnaires

| Order* | Measurement | Patients  at baseline | Caregivers  at baseline | Patients  at 3 months later |
| --- | --- | --- | --- | --- |
| 1 | Demographic characteristics | ✓ | ✓ |  |
| 2 | Quality of life** | ✓ |  | ✓ |
| 3 | Perceptions of therapy goals | ✓ |  | ✓ |
| 4 | Perceptions of oncologist disclosure of incurability | ✓ | ✓ | ✓ |
| 5 | Perceptions of illness | ✓ | ✓ | ✓ |
| 6 | Preference for future medical treatment | ✓ | ✓ | ✓ |
| 7 | Physician compassion | ✓ |  |  |
| 8 | Depressive symptoms*** | ✓ | ✓ | ✓ |

*Order in which the questionnaires were presented

**Quality of life was measured using the Comprehensive Quality of Life Outcome questionnaire

***Depressive symptoms were measured using the Patient Health Questionnaire - 9
