# Supplementary figures and images for: Prognostic Awareness and Discussions of Incurability in Patients with Pretreated Non-Small Cell Lung Cancer and Caregivers: A Prospective Cohort Study
Source: Oncologist. 2022 Sep 6;27(11):982–90. doi: 10.1093/oncolo/oyac178 (PMC9632306; doi:10.1093/oncolo/oyac178)

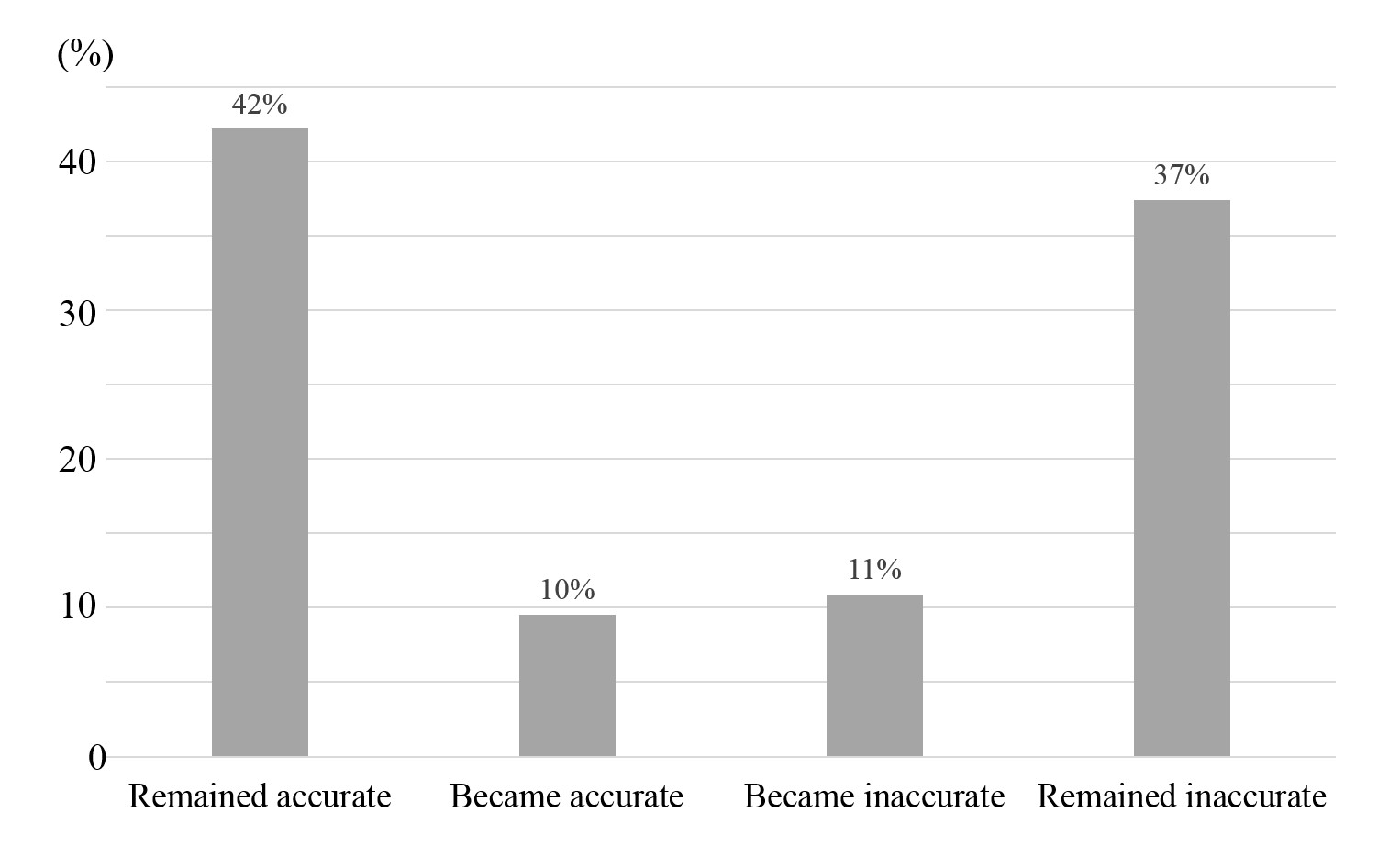

Supplement: oyac178_suppl_Supplementary_Figure_S1 [file oyac178_suppl_supplementary_figure_s1.jpeg]

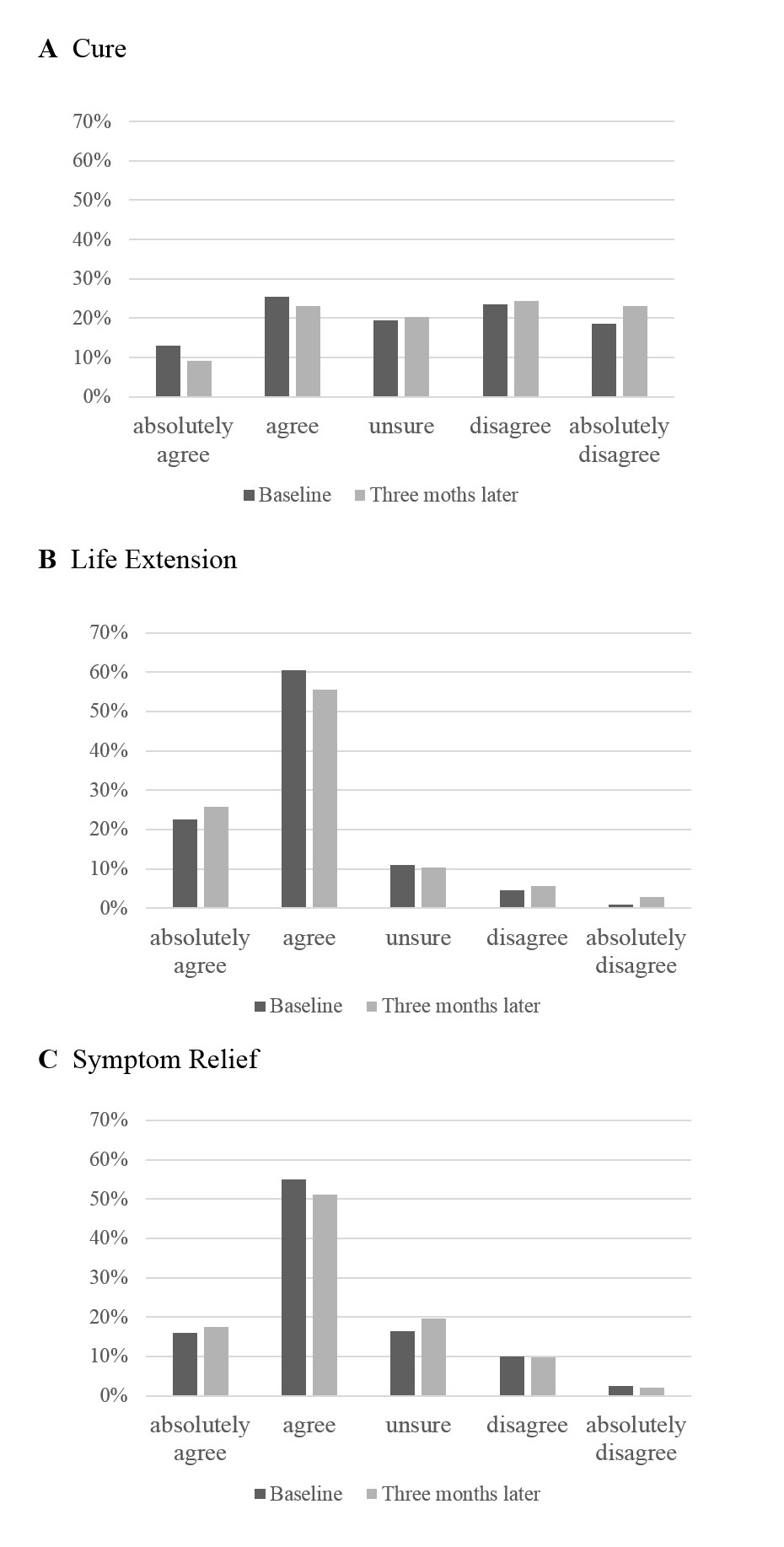

Supplement: oyac178_suppl_Supplementary_Figure_S2 [file oyac178_suppl_supplementary_figure_s2.jpeg]
